# Editorial Notes, News and Miscellany

**Published:** 1914-03

**Authors:** 


### Editorial Notes, News and Miscellany.

A SAMPLE COPY means "your subscription is solicited." I also solicit papers for publication.

---

HONORABLE MEN pay for their journals. Only the deadbeats do not. "I've got a little list."

---

A DOCTOR IN NEW YORK has discovered a cure for cigarette habit, and is said to be practicing it successfully. He cauterizes the throat with nitrate of silver. Try it!

---

DR. M. M. CARRICK, of Dallas, by the splendid work he has done and is doing in sanitation and hygiene, has demonstrated a masterly grasp of the subject, which entitles him to favorable consideration under next administration for the office of State Health Officer. The State should command its highest talent in every department.

The Mississippi Legislature has raised "the age of consent" to 18 years. Two hundred club women besieged them and never let up until they secured the passage of the bill.

---

Dr. C. C. Walker, of Gainesville, Texas, died in California, February 24th (ult.), aged 70. Dr. Walker had practiced medicine in Gainesville more than a quarter of a century, and was universally respected and esteemed. His health failed about two years ago, and gave up practice and went to California, in the hope of regaining it. He was a Confederate veteran.

---

Neither.—Last month I inquired in this department: Do you "operate" a case, or operate *on* one? I want to know. I observe that some of our best surgeons, in reporting cases, say they "operated" them.

Dr. Arthur E. Sweatland, of Nacogdoches, Texas, suggests that we say "operated upon." Correct.

---

My friend, Dr. Matthew M. Smith, editor and owner of the *Texas Medical News*, at Dallas, is publishing a most excellent journal. It gets better all the time, and so does he. Always handsome, he gets better looking every day. Well, he deserves to succeed at everything, for he is a most excellent gentleman, an accomplished physician and a genial and delightful companion, socially. Moreover, he is as full of energy as a buzz-saw, and is always on the go. I hope everybody who reads this will send a postal card for a sample copy of the *Medical News*, and next mail send a one dollar bill. It will be about the biggest dollar's worth, ever. Dr. Smith is Medical Director of the Praetorians of America.

---

Incidental Case.—Dr. Rubetinker was a qualified M. D., but, settling in a cattle country and finding the demand strong, he had added veterinary work to his other practice.

"Nothing serious," announced the doctor, after examining a valuable bull which he had been summoned post-haste to treat. "Give him one of these powders in a quart of bran mash three times a day."

The rancher heaved a sight of relief. "Wait," he said, as the M. D., V. S., was about to leave. "I reckon, as long as you're here, you might as well have a look at the old woman. She's been ailin' for a month or two."—*Judge.*

THE PAID-UP SUBSCRIBER.—"Let this sink into your soul."

How dear to our heart is the steady subscriber
Who pays in advance at birth of each year,
Who lays down the money and does it quite gladly,
And casts 'round the office a halo of cheer.
He never says, "Stop it; I cannot afford it,
I'm getting more journals than now I can read."

But always says, "Send it; we doctors all like it—
In fact we all think it a help and a need."
How welcome his check when it reaches our sanctum,
How it makes our pulse throb; how it makes our heart dance.
We outwardly thank him; we inwardly bless him—
The steady subscriber who pays in advance.

—*Medical Herald.*

---

If you think you're beaten, you are,
If you think you dare not, you don't,
If you'd like to win, but think you can't
It's almost a cinch you won't.
If you think you'll lose, you've lost,
For out in the world you will find
Success begins with a fellow's will,
It's all in the state of his mind.

Full many a race is lost
Ere ever a step is run;
And many a coward fails
Ere ever his work's begun.
Think big and your deeds will grow,
Think small, and you'll fall behind,
Think that you can and you will,
It's all in the state of the mind.

If you think you're outclassed, you are;
You've got to think high to rise,
You've got to be sure of yourself before
You can ever win a prize.
Life's battles don't always go
To the stronger or faster man,
But soon or late the man who wins
Is the fellow who thinks he can.

—*J. Phileas Fogg.*

